# Differences in Fecal Microbiome and Antimicrobial Resistance between Captive and Free-Range Sika Deer under the Same Exposure of Antibiotic Anthelmintics

**DOI:** 10.1128/Spectrum.01918-21

**Published:** 2021-12-01

**Authors:** Kangqi Wu, Yongtao Xu, Weiwei Zhang, Huirong Mao, Biao Chen, Yunlin Zheng, Xiaolong Hu

**Affiliations:** a College of Animal Science and Technology, Jiangxi Agricultural University, Nanchang, China; b College of Forestry, Jiangxi Agricultural University, Nanchang, China; University of Minnesota

**Keywords:** antimicrobial resistance, fecal microbiome, sika deer, antibiotics, microbial metagenomics

## Abstract

This study aimed to compare the fecal microbiome and antimicrobial resistance between captive and free-range sika deer with the same exposure to antibiotic anthelmintics. The taxonomic differences mainly involved significant changes in the dominant phyla, genera, and species. Linear discriminant analysis effect size (LEfSe) analysis revealed that 22 taxa were significantly different between the two groups. The KEGG analysis showed that the fecal microbiome metabolic function, and all level 2 categories in metabolism had higher abundance in the free-range deer. Based on the carbohydrate-active enzyme (CAZy) database analysis, glycoside hydrolases and carbohydrate-binding modules showed remarkable differences between the two groups. Regarding antibiotic resistance, *tet*Q and *lnu*C dominated the antibiotic resistance ontology (ARO) terms, and tetracycline and lincosamide resistance dominated the antimicrobial resistance patterns. Furthermore, the *lnu*C, *Erm*F, and *tet*W/N/W AROs and lincosamide resistance showed higher abundance in the captive deer, suggesting that captivity may yield more serious resistance issues because of the differences in greenfeed diet, breeding density, and/or housing environment. The results also revealed important associations between the phylum *Proteobacteria*, genus *Prevotella*, and major antibiotic resistance genes. Although the present study was a pilot study with a limited sample size that was insufficient control for some potential factors, it serves as the metagenomic study on the microbial communities and antimicrobial resistance in sika deer.

**IMPORTANCE** We used a metagenomic approach to investigate whether and how captive and free-range impact the microbial communities and antimicrobial resistance in sika deer. The results provide solid evidence of the significant impacts on the microbial composition and function in captive and free-range sika deer. Interestingly, although the sika deer had the same exposure to antibiotic anthelmintics, the antimicrobial resistances were affected by the breeding environment.

## INTRODUCTION

The wild population of sika deer (*Cervus nippon*) in China is first-grade state-protected animals. Among the four subspecies (*C.n. hortulorum*, *C.n. taiouanus*, *C.n. sichuanicus*, and *C.n. kopschi*) remaining in China (1), only *C.n. hortulorum* is subject to large-scale, farmed breeding. The population of cultivated sika deer in China is now more than 550,000 (2). The “National Breed List of Livestock and Poultry Genetic Resources” was issued in 2020 and updated in 2021 by the Chinese government. It lists one indigenous sika deer breed (Jilin sika deer) and 7 cultivated sika deer breeds (Siping, Aodong, Dongfeng, Xingkaihu, Shuangyang, Xifeng, and Dongda sika deer) as the main special livestock. The favorable policies and booming market have promoted the large-scale intensive farming of sika deer in China. In southern China, more and more sika deer farms have been established, some of which mainly use a free-range approach because of their rich plant resources. Therefore, it is necessary to investigate the difference in the gut microbiota of captive and free-range sika deer.

With the population of farmed sika deer increasing, parasite infection and other diseases have become important issues (3). More and more anthelmintics (mainly antibiotics) and antimicrobials are being used to prevent and treat animal diseases (4), which has profound effects on indigenous microbes in animal feces (5). Animal fecal microbiota represents a vast reservoir of antibiotic resistance genes (ARGs) (6), and the antibiotic residues, resistant bacteria, and ARGs in feces can be transported into the environment via manure application, leakage, runoff, and airborne particulate matter (7, 8), adding to the serious global issue regarding the effects of antibiotic resistance on animal and human health. Ivermectin (e.g., in fenbendazole-ivermectin tablets and albendazole-ivermectin powder) is one of the most used antibiotic deworming drugs used in sika deer management. However, few studies on sika deer have explored the impacts of antibiotics on the bacterial resistome in the gastrointestinal tract and resultant feces. Furthermore, it is unknown whether antibiotic resistance in the fecal microbiome of sika deer with the same exposure of antibiotic anthelmintics differs with different rearing conditions. This is an important criterion when evaluating the advantages and disadvantages of captive and free-range sika deer.

Current studies on the gut microbiome in sika deer focus on basic biology and growth performance. Guan et al. (2) compared the gut microbiota between wild and captive sika deer, and Li et al. (9) revealed the intestinal microbiota and metabolome from birth to weaning. Li et al. (10, 11) investigated the effects of tannin and diets on the rumen microbiota and found that diet-induced changes in the gut microbiota are associated with growth performance. Our recent study (12) investigated the effects of antibiotic treatment (fenbendazole-ivermectin tablets) on the gut bacterial and fungal communities of domesticated sika deer, and we found substantial differences after treatment. However, the antibiotic-induced changes in ARGs in the fecal microbiome still need to be clarified.

Next-generation sequencing-based metagenomic approaches allow comprehensive exploration of uncultivable and rare taxa from the immense diversity in the fecal microbial population. This technology has been widely used to analyze microbial communities (13, 14) and conduct qualitative and quantitative ARG analyses based on animal feces (15, 16). It is necessary to investigate the ARGs and bacterial communities in the feces of sika deer, which represent a growing animal husbandry industry in China. Furthermore, it is important to verify the responses of ARG-harboring bacteria in hosts with antibiotic exposure.

This study used metagenomic sequencing to profile the impacts on the fecal resistome and microbiota of captive and free-range sika deer with the same routine exposure of antibiotic anthelmintics. The results will be helpful to understand the potential dysbiosis resulting from antibiotic treatment, and to develop optimal sika deer feeding and parasite control measures.

## RESULTS

### Overview of data.

After processing the raw reads, a total of 76347.95 Mbp of clean data was obtained from the 12 fecal samples of sika deer. The mean clean data per sample comprised 6362.33 ± 268.14 Mbp, with a range of 6003.94 to 6839.32 Mbp (Table S1). *De novo* metagenomic assembly based on the 12 fecal samples resulted in 1,456,305 scaftigs, with each sample ranging from 98,224 to 141,424 (mean = 121,359 ± 12,423; Table S2). These scaftigs had a mean length in the samples of 1121.78 to 1355.90 bp (overall mean = 1238.29 ± 59.22 bp), and the *N*_50_ sequence length ranged from 1167 to 1614 bp (mean = 1390 ± 114 bp; Table S2). There were 2,617,193 (mean = 218,099 ± 24,637) predicted open reading frames (ORFs) in the 12 samples (Table S3). The total length of ORFs per sample ranged from 106.34 to 156.59 Mbp (mean = 134.88 ± 16.42 Mbp), and the mean ORF length ranged from 594.91 to 650.09 bp (mean = 617.98 ± 15.66 bp, Table S3). The within and between-group Bray–Curtis similarities were exhibited in Table S4, and these similarities showed no significant difference (analysis of variance, F = 1.76, *P = *0.18).

### Taxonomic analysis of fecal microbiome.

**FIG 1:**
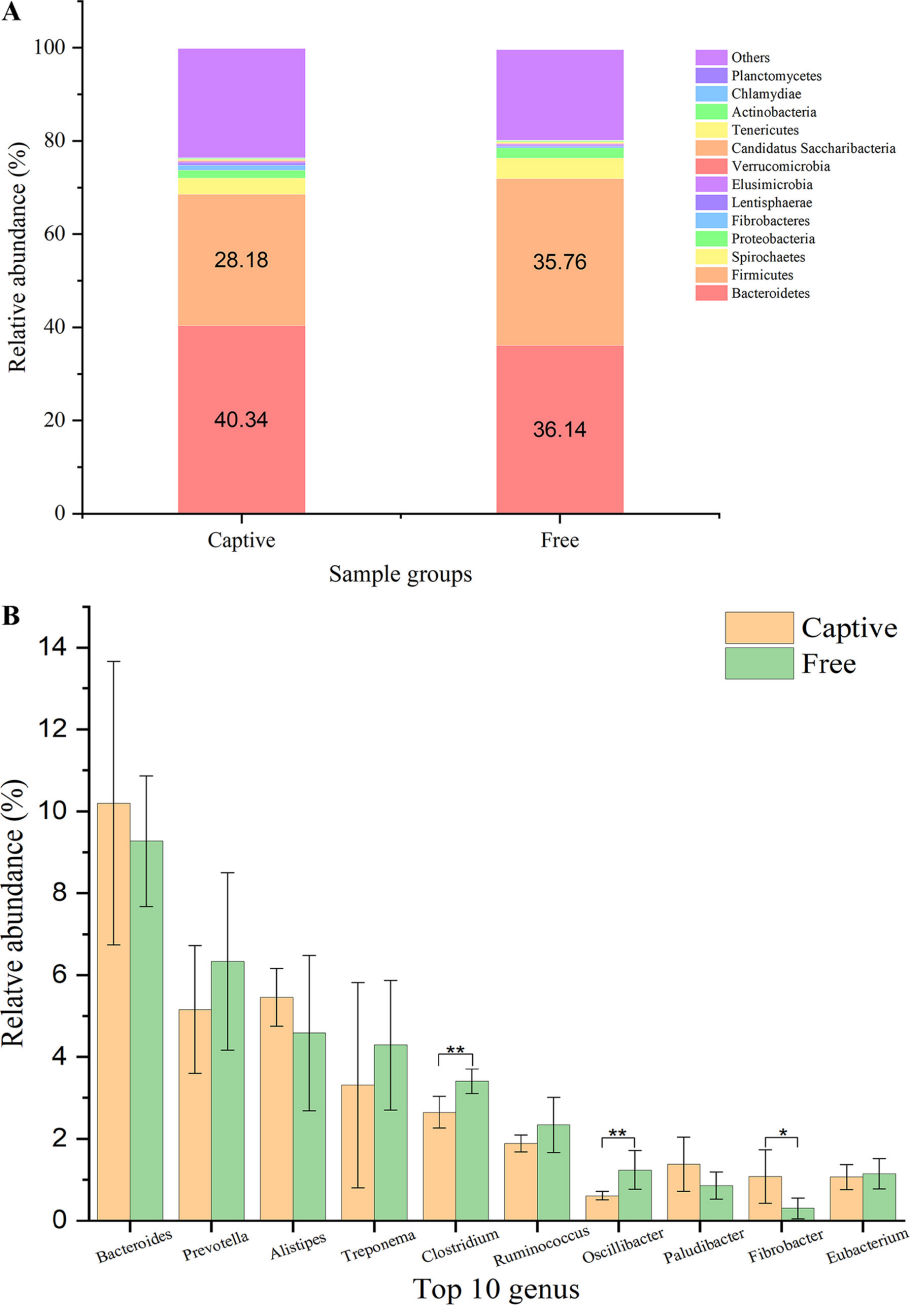
Relative abundance of the fecal microbiome at the phyla level (A) and the genus level (B) in captive (Captive) and free-range (Free) sika deer. (A) The fecal microbial composition based on the mean values of relative abundance of each phylum, the sequences with low mean relative abundance (<0.1%) were assigned as “Others”. The number in the histogram is the relative abundance (%) of *Bacteroidetes* and *Firmicutes*. (B) The comparisons of top 10 genera. *, *P < *0.05; **, *P < *0.01.

At the phylum level, 75.26% of sequences could be annotated. The taxonomic composition of the 12 samples at the phylum level (with relative abundance ≥0.1%) is shown in Fig. S1. The top 10 phyla were *Bacteroidetes*, *Firmicutes*, *Spirochetes*, *Proteobacteria*, *Fibrobacteres*, *Lentisphaerae*, *Elusimicrobia*, *Verrucomicrobia*, *Candidatus Saccharibacteria*, and *Tenericutes*. *Bacteroidetes* and *Firmicutes* overwhelmingly dominated the fecal microbiome in both groups (Fig. 1A). Among the top 10 phyla, *Firmicutes* had a significantly higher abundance in the free-range deer (*t* test, *t* = 4.43, *P < *0.01), while *Fibrobacteres* had a significantly higher abundance in the captive deer (*t* test, *t* = 2.59, *P = *0.03; Table 1). Additionally, the *Firmicutes*/*Bacteroidetes* (F/B ratio) was significantly higher in the free-range deer than the captive deer (*t* test, *t* = 3.49, *P < *0.01; Table 1). The top 10 genera belonged to the phyla *Bacteroidetes* (*Bacteroides*, *Prevotella*, *Alistipes*, and *Paludibacter*), *Spirochetes* (Treponema), *Firmicutes* (*Clostridium*, *Ruminococcus*, *Oscillibacter*, and *Eubacterium*), and *Fibrobacteres* (*Fibrobacter*). *Clostridium* (*t* test, *t* = 3.83, *P < *0.01) and *Oscillibacter* (*t* test, *t* = 3.18, *P = *0.01) had a significantly higher abundance in the free-range deer, whereas *Fibrobacter* (*t* test, *t* = 2.70, *P = *0.02) had a significantly higher abundance in the captive deer (Fig. 1B). The top 10 species were *Firmicutes bacterium CAG:110*, Treponema porcinum, Treponema bryantii, *Bacteroidales bacterium 52_46*, Treponema brennaborense, Paludibacter propionicigenes, Ruminococcus flavefaciens, Ruminococcus bromii, *Prevotella* sp. *CAG:279*, and *Clostridium* sp. *CAG:448*. *Firmicutes bacterium CAG:110* (*t* test, *t* = 2.70, *P = *0.02) had a significantly higher abundance in the free-range deer than the captive deer.

**TABLE 1:** The comparison of relative abundance (mean ± SD) of top 10 phyla and *Firmicutes*/*Bacteroidetes* ratio (F/B ratio) in fecal microbiome of captive (Captive) and free-range (Free) sika deer

Top 10 phyla	Captive	Free	Statistic value
*Bacteroidetes*	40.34 ± 4.71	36.14 ± 2.35	t = 1.95, *P* = 0.08^*a*^
*Firmicutes*	28.18 ± 2.12	35.76 ± 3.61	t = 4.43, *P* < 0.01^*a*^
F/B ratio	0.71 ± 0.14	1.00 ± 0.15	t = 3.49, *P* < 0.01^*a*^
*Spirochetes*	3.48 ± 2.55	4.44 ± 1.59	t = 0.78, *P* = 0.45^*a*^
*Proteobacteria*	1.67 ± 1.16	2.13 ± 2.01	U = 16.00, *P* = 0.82^*b*^
*Fibrobacteres*	1.09 ± 0.66	0.32 ± 0.30	t = 2.59, *P* = 0.03^*a*^
*Lentisphaerae*	0.51 ± 0.46	0.27 ± 0.27	t = 1.14, *P* = 0.28^*a*^
*Elusimicrobia*	0.27 ± 0.34	0.24 ± 0.32	U = 14.00, *P* = 0.59^*b*^
*Verrucomicrobia*	0.22 ± 0.22	0.10 ± 0.08	t = 1.17, *P* = 0.27^*a*^
*Candidatus Saccharibacteria*	0.01 ± 0.01	0.12 ± 0.23	U = 14.00, *P* = 0.52^*b*^
*Tenericutes*	0.30 ± 0.15	0.31 ± 0.10	t = 0.09, *P* = 0.93^*a*^

a The significances were determined by Student's *t* test.

b The significances were determined by the Mann-Whitney U test.

**FIG 2:**
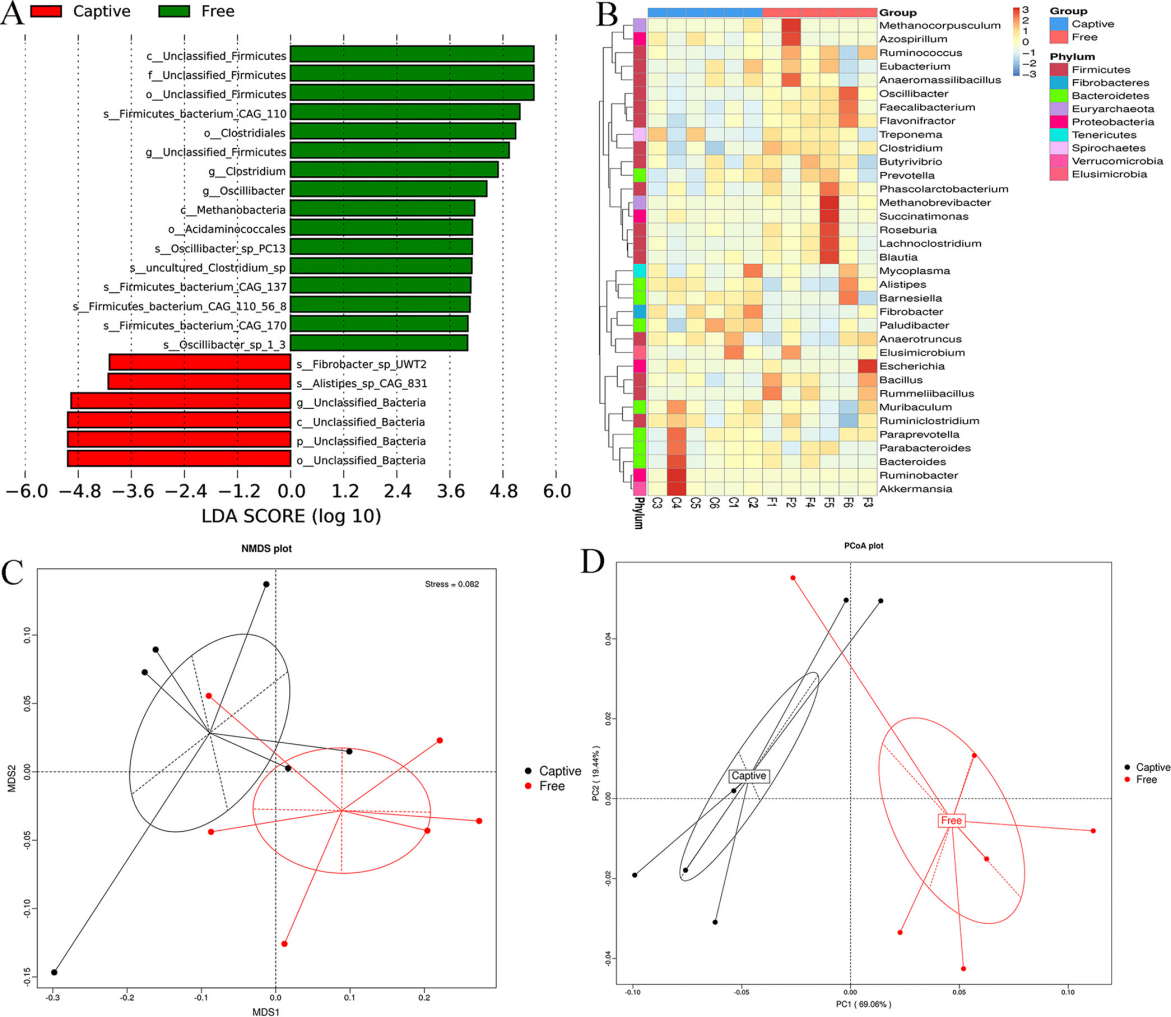
A combination of linear discriminant analysis (LDA) effect size (LEfSe) analysis (A; LDA score is 3), analysis of similarities (B; ANOSIM, based on the relative abundance of phyla), Nonmetric multidimensional scaling plots (C; NMDS, based on Bray-Curtis dissimilarities) and principal coordinate analysis plots (D; PCoA, based on weighted Unifrac distances) represents the taxonomic differences and distribution among the samples and between captive (Captive) and free-range (Free) sika deer.

The linear discriminant analysis (LDA) effect size (LEfSe) analysis revealed that 22 microbial taxa were significantly different between the two groups, comprising 1 phylum, 3 classes, 4 orders, 1 family, 4 genera, and 9 species (Fig. 2A). Although the nonmetric multidimensional scaling (NMDS) plots showed some overlap among the 12 samples between the captive and free-range groups (Fig. 2C), the principal coordinate analysis (PCoA) plot exhibited divergent clusters between the two groups (Fig. 2D). Furthermore, the analysis of similarities (ANOSIM) based on the relative abundance of phyla revealed significant differences between the two groups (ANOSIM, *R* = 0.54, *P < *0.01; Fig. 2B).

### Functional analysis of fecal microbiomes.

**FIG 3:**
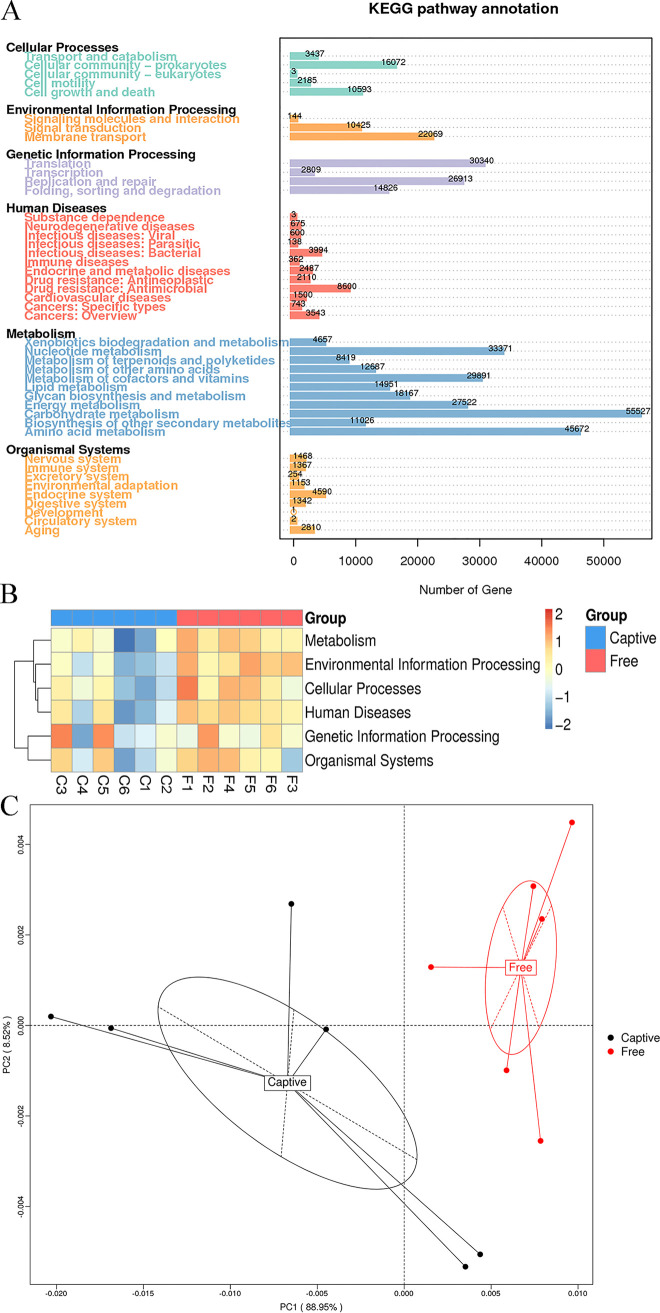
The function of the fecal microbiome in sika deer based on the KEGG database. (A) The abundance of the functional pathway at level 1 and level 2 categories. Heatmap analysis (B) and principal coordinate analysis plots (C; PCoA, based on Bray-Curtis dissimilarities) based on the abundance of level 1 functional categories shows the differences in the function of the fecal microbiome in captive (Captive) and free-range (Free) sika deer.

A total of 1,442,324 UniGenes were obtained from the 12 samples. Of these, 813,720 (56.42%) were annotated using the Kyoto encyclopedia of genes and genomes (KEGG) database. A total of 472,322 UniGenes (32.75%) were assigned to 5,157 KEGG ortholog groups (KOs), and 278,648 UniGenes (19.32%) were assigned to 276 KEGG pathways. Additionally, 291,997 UniGenes (20.24%) were annotated to 1,793 KEGG enzymes (identified by enzyme commission (EC) numbers). Among the 6 level 1 KEGG functional categories, metabolism dominated the fecal microbiome function (59.46%), followed by genetic information processing (17.00%), environmental information processing (7.41%), cellular processes (7.33%), human diseases (5.85%), and organismal systems (2.95%; Fig. 3A). The dominant level 2 KEGG categories were carbohydrate metabolism, amino acid metabolism, nucleotide metabolism, metabolism of cofactors and vitamins, energy metabolism, translation, and replication and repair (Fig. 3A).

**FIG 4:**
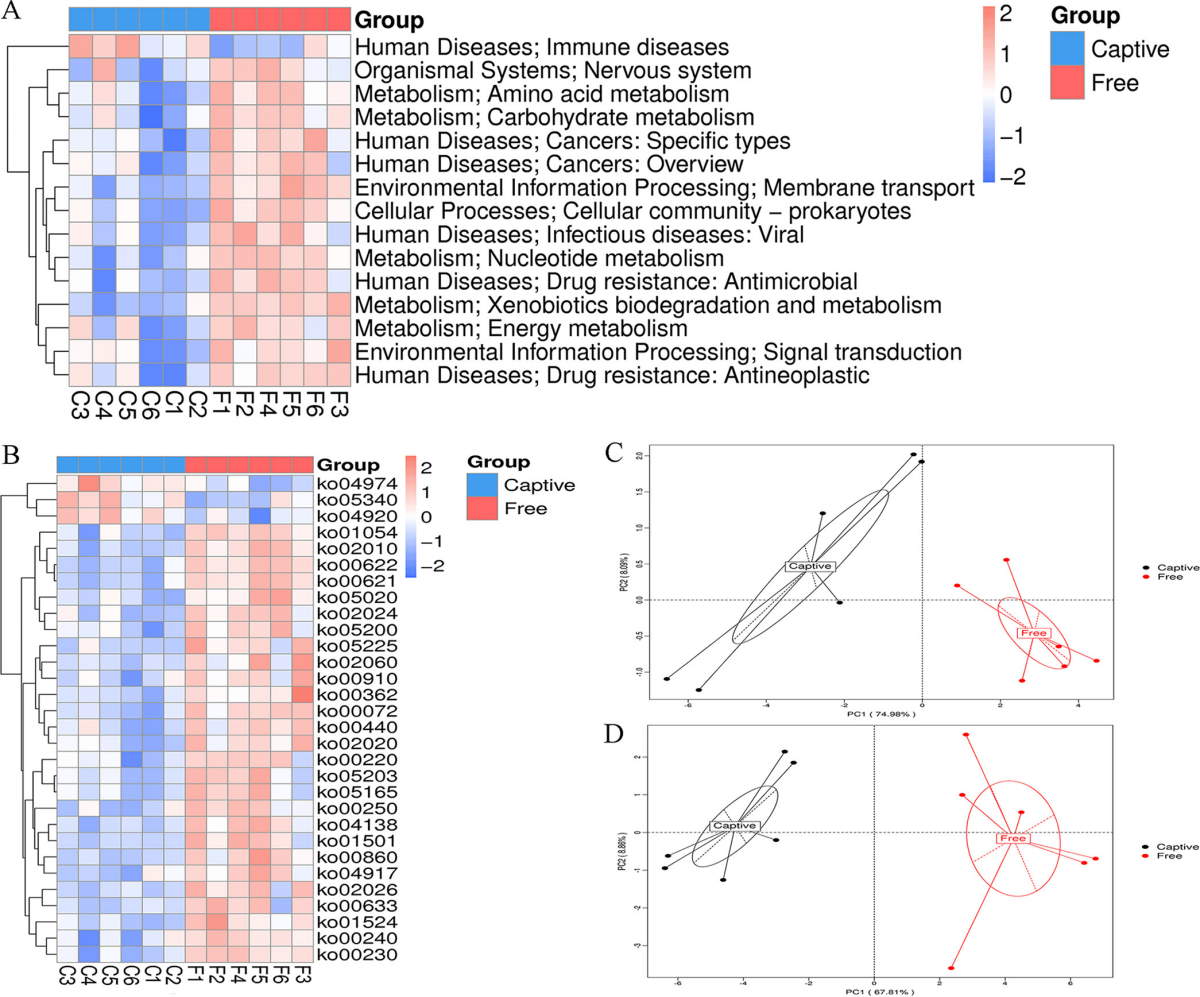
The Metastats analysis of KEGG level 2 and level 3 functional categories between captive (Captive) and free-range (Free) sika deer. (A) 15 KEGG level 2 categories were significantly different between the two groups. (B) 30 level 3 categories were significantly different between the two groups. Principal coordinate analysis plots (based on Bray-Curtis dissimilarities) based on the abundance of level 2 (C) and level 3 (D) functional categories showed the differences in the function of the fecal microbiome in captive (Captive) and free-range (Free) sika deer.

Among the 6 level 1 KEGG categories, metabolism (Mann-Whitney test, U = 2.72, *P < *0.01), environmental information processing (*t* test, *t* = 5.65, *P < *0.01), cellular processes (*t* test, *t* = 2.90, *P = *0.02), and human diseases (Mann-Whitney test, U = 2.24, *P = *0.03) were significantly higher in the free-range deer than the captive deer (Table 2). The PCoA plot based on the abundance of level 1 categories revealed that the six samples in each group clustered into two groups (Fig. 3C). The heatmap confirmed the significant differences (Fig. 3B). ANOSIM indicated statistical significance between the two groups (ANOSIM, *R* = 0.44, *P < *0.01). The Metastats analysis found that 15 level 2 (Fig. 4A) and 30 level 3 (Fig. 4B) KEGG categories were significantly different between the two groups, and the PCA plots showed obvious differences (Fig. 4C and D). Noticeably, among the level 2 categories, all the metabolic categories, comprising amino acid metabolism, carbohydrate metabolism, nucleotide metabolism, xenobiotics biodegradation and metabolism, and energy metabolism, showed higher abundance in the free-range group (Fig. 4A).

**TABLE 2:** The comparison of relative abundance (mean ± SD) of KEGG level 1 functional categories in fecal microbiome of captive (Captive) and free-range (Free) sika deer

Level 1 categories	Captive	Free	Statistic value
Metabolism	13.30 ± 0.48	13.93 ± 0.19	U = 2.72, *P* < 0.01^*a*^
Genetic information processing	6.27 ± 0.39	6.33 ± 0.22	U = 0.32, *P *= 0.82^*a*^
Environmental information processing	2.17 ± 0.13	2.52 ± 0.08	t = 5.65, *P* < 0.01^*b*^
Cellular processes	1.89 ± 0.12	2.08 ± 0.10	t = 2.90, *P* = 0.02^*b*^
Human diseases	1.54 ± 0.07	1.64 ± 0.02	U = 2.24, *P* = 0.03^*a*^
Organismal systems	0.86 ± 0.04	0.89 ± 0.04	t = 1.26, *P* = 0.24^*b*^

a The significances were determined by Mann-Whitney U test.

b The significances were determined by Student's *t* test.

**FIG 5:**
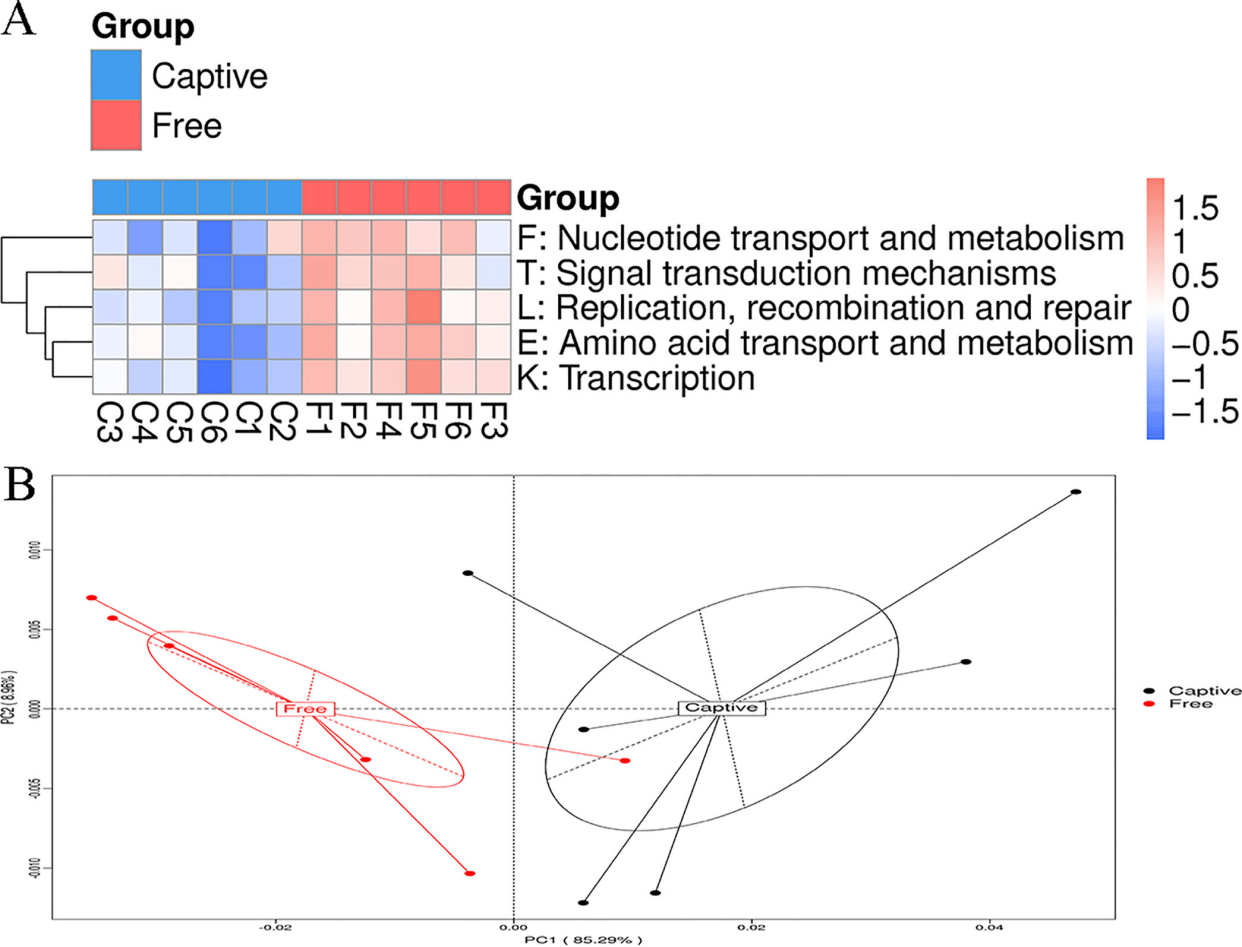
The function of the fecal microbiome in sika deer based on the eggNOG database. Heatmap analysis (A) and Principal coordinate analysis plots (B; PCoA, based on Bray-Curtis dissimilarities) based on the abundance of level 1 functional categories shows the differences in the function of the fecal microbiome in captive (Captive) and free-range (Free) sika deer.

When the UniGenes were subjected to a basic local alignment search tool (BLAST) search against the evolutionary genealogy of genes: Non-supervised Orthologous Groups (eggNOG) database, 803,425 UniGenes (55.70%) were annotated to 17,368 ortholog groups (OGs). The significantly different level 1 functional categories identified by the Metastats analysis were “replication, recombination and repair”, “amino acid transport and metabolism”, “transcription”, “nucleotide transport and metabolism”, and “signal transduction mechanisms” (Fig. 5A). The PCoA plot showed distinct clustering between the two groups (Fig. 5B), and ANOSIM verified the significant difference (ANOSIM, *R* = 0.34, *P = *0.02).

Using the carbohydrate-active enzymes (CAZy) database, 52,333 UniGenes (3.63%) were annotated. The most dominant CAZymes were glycoside hydrolases (GHs; 31789, 57.43%). A total of 316 CAZymes were identified, comprising 151 GHs, 77 glycosyltransferases (GTs), 48 carbohydrate-binding modules (CBMs), 27 polysaccharide lyases (PLs), 9 carbohydrate esterases (CEs), and 4 auxiliary activities (AAs). The top 10 CAZymes were GH2, GT2, GH13, GH43, GH3, GH20, GT4, CBM48, GH92, and GH78 (Fig. S2). The relative abundance of CBM48 was significantly higher in the free-range deer than in the captive deer (*t* test, *t* = 2.23, *P = *0.05). The Metastats analysis also revealed that 38 CAZymes (18 GHs, 9 CBMs, 6 GTs, 3 CEs, and 2 PLs) were significantly different between the two groups (Metastats, *P < *0.05).

### Antibiotic resistance in the fecal microbiome.

**FIG 6:**
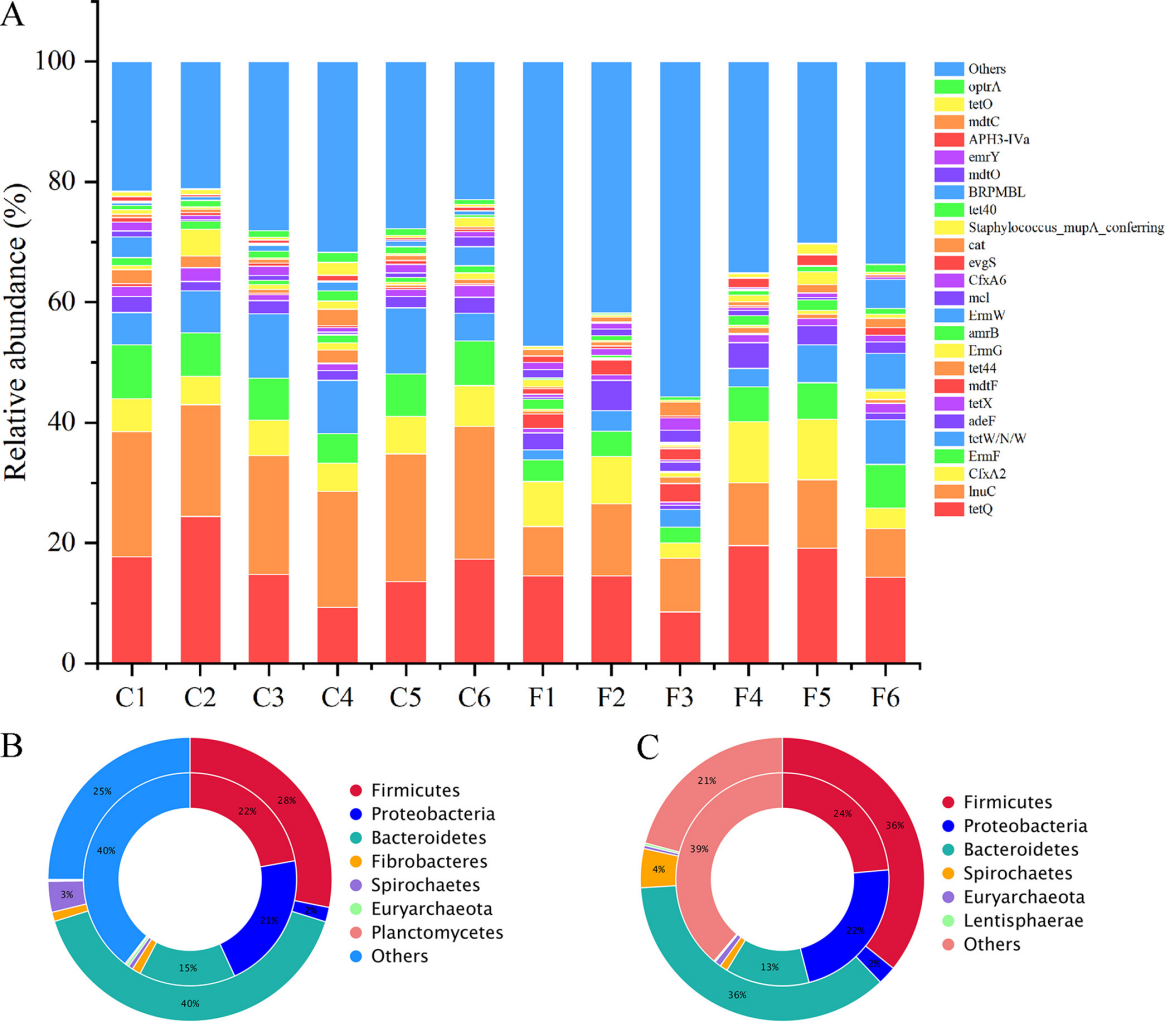
The relative abundance of antibiotic resistance ontologies (AROs) in the fecal microbiome of each animal (A) and the Circos plots of the phylogenetic composition of AROs and microbial communities at the phyla level for captive deer (B) and free-range deer (C). The inner cycle represents the distribution of AROs in corresponding microbial phyla, and the outer cycle represents the overall distribution of the fecal microbial community.

A total of 508 UniGenes were annotated using the Comprehensive Antibiotic Resistance Database (CARD), and 330 antibiotic resistance ontology terms (AROs) were identified from the 12 fecal samples. The main AROs in each sample are shown in Fig. 6A. The top 10 AROs were *tet*Q, *lnu*C, *Cfx*A2, *tet*W/N/W, *Erm*F, *mdt*F, *ade*F, *mdt*C, *Erm*W, and *emr*Y. Among the top 10 AROs, the abundance of *lnu*C, *Erm*F, and *tet*W/N/W were significantly higher in the captive group than in the free-range group (Table 3). The phyla *Firmicutes*, *Proteobacteria*, and *Bacteroidetes* were dominant antibiotic resistance microbial communities at phylum level in both captive (Fig. 6B, inner cycle) and free-range groups (Fig. 6C, inner cycle). In particular, the proportion of *Proteobacteria* in the antibiotic resistance microbial communities was higher than in the total microbial community, suggesting that *Proteobacteria* harbors a greater proportion of resistance genes. Nevertheless, the NMDS plot based on the Bray-Curtis distance of the relative abundance of the AROs revealed no significantly distinct distributions between the two groups (Fig. S3), and the corresponding ANOSIM confirmed the NMDS plot (*R* = 0.04, *P = *0.26).

**TABLE 3:** The comparison of relative abundance (mean ± SD) of top 10 antibiotic resistance ontologies (AROs) in fecal microbiome of captive (Captive) and free-range (Free) sika deer

AROs	Captive	Free	Statistic value
*tetQ*	16.21 ± 5.05	15.12 ± 4.00	t = 0.41, *P* = 0.69^*a*^
*lnuC*	20.25 ± 1.30	9.84 ± 1.68	t = 12.02, *P* < 0.01^*a*^
*CfxA2*	5.65 ± 0.83	6.89 ± 3.25	t = 0.91, *P* = 0.38^*a*^
*ErmF*	7.07 ± 1.30	4.93 ± 1.73	t = 2.43, *P* = 0.03^*a*^
*tetW/N/W*	7.91 ± 2.69	4.13 ± 2.23	t = 2.65, *P* = 0.02^*a*^
*adeF*	2.09 ± 0.51	2.87 ± 1.70	t = 1.08, *P* = 0.31^*a*^
*tetX*	1.54 ± 0.51	1.02 ± 0.40	t = 1.96, *P* = 0.08^*a*^
*mdtF*	0.24 ± 0.11	1.37 ± 1.43	U = 0.32, *P* = 0.82^*b*^
*tet44*	1.38 ± 0.86	0.70 ± 0.30	U = 1.12, *P* = 0.31^*b*^
*ErmG*	1.44 ± 1.49	0.59 ± 0.45	U = 1.60, *P* = 0.13^*b*^

a The significances were determined by Student's *t* test.

b The significances were determined by the Mann-Whitney U test.

**FIG 7:**
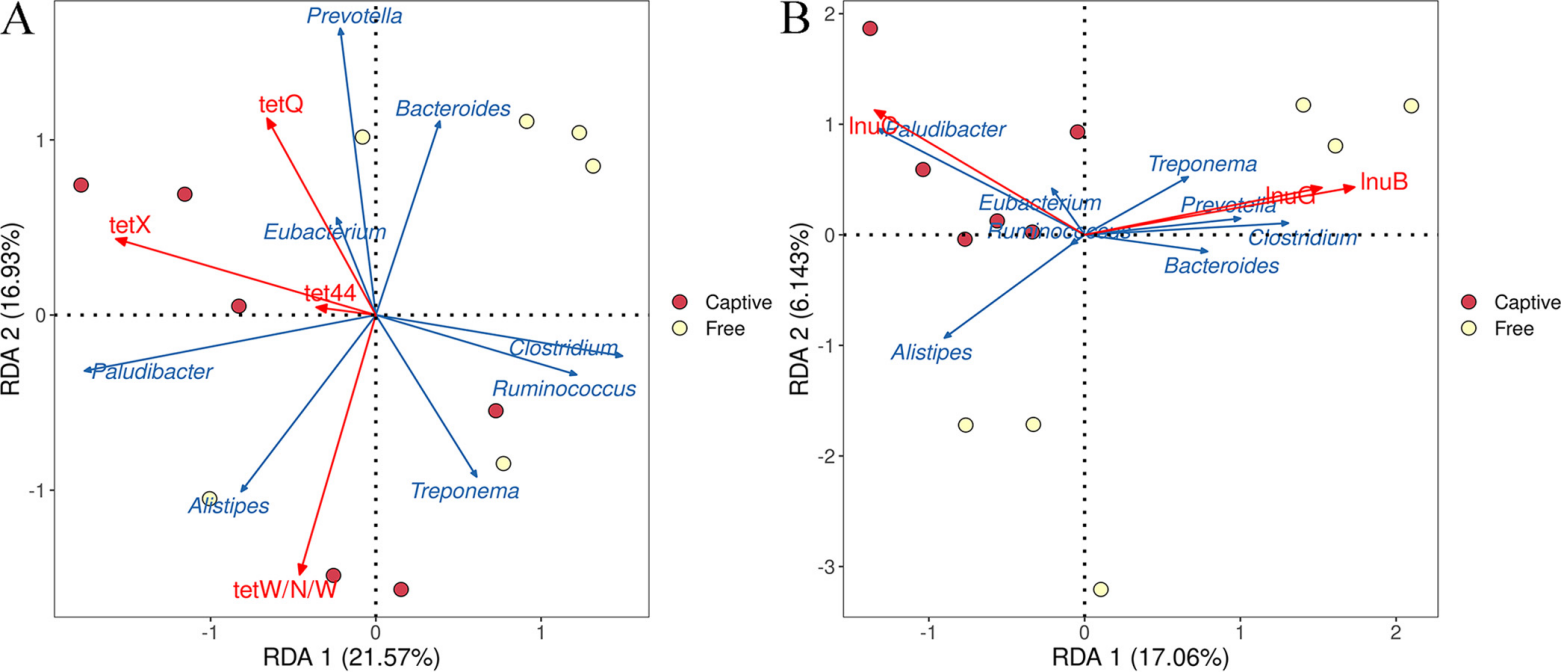
Redundancy analysis (RDA) plots showing the distribution of captive (Captive) and free-range (Free) sika deer raised under similar antibiotic exposure, microbiome genera, and antimicrobial resistance (AMR) genes according to corresponding antimicrobial drug resistance patterns. (A) RDA plot of AMR genes corresponding to tetracycline drug resistance pattern. (B) RDA plot of AMR genes corresponding to lincosamide drug resistance pattern.

The antimicrobial resistance patterns were determined by analyzing the ARGs. Tetracycline and lincosamide resistance dominated the drug resistance patterns in both groups, and the other top 10 antimicrobial resistance patterns were macrolide and lincosamide, streptogramin, cephamycin, aminoglycoside, fluoroquinolone and tetracycline, glycopeptide antibiotics, peptide antibiotics, phenicol, macrolide and fluoroquinolone, and penam resistance (Fig. S4). Among these, the abundance of lincosamide resistance was significantly higher in the captive group (*t* test, *t* = 11.22, *P < *0.01), whereas the abundance of aminoglycoside resistance was significantly higher in the free-range group (*t* test, *t* = 4.55, *P < *0.01; Table 4). Nonparametric permutation tests in the redundancy analysis (RDA) showed that tetracycline and lincosamide resistance were significantly different between the two groups. The RDA of tetracycline resistance showed that *tet*Q, *tet*X, and *tet*W/N/W in the captive group were related to the dominant genera *Prevotella*, *Eubacterium*, *Paludibacter*, and *Alistipes*, but no tetracycline resistance AROs were related to the dominant genera in the free-range group (Fig. 7A). The RDA of lincosamide resistance showed that *lnu*C in the captive group was related to *Paludibacter* and *Eubacterium*, and *lnu*B and *lnu*G in the free-range group were related to *Prevotella*, *Clostridium*, Treponema, and *Bacteroides* (Fig. 7B).

**TABLE 4:** The comparison of relative abundance (mean ± SD) of top 10 antimicrobial resistance (AMRs) in fecal microbiome of captive (Captive) and free-range (Free) sika deer

AMRs	Captive	Free	Statistic value
Tetracycline	28.49 ± 4.11	23.30 ± 5.11	t = 1.94, *P *= 0.08^*a*^
Lincosamide	20.95 ± 1.44	10.56 ± 1.75	t = 11.22, *P* < 0.01^*a*^
Macrolide, lincosamide, streptogramin	10.38 ± 3.13	7.17 ± 3.91	t = 1.57, *P *= 0.15^*a*^
Cephamycin	6.82 ± 1.08	7.69 ± 3.25	t = 0.62, *P *= 0.55^*a*^
Aminoglycoside	5.55 ± 0.69	8.71 ± 1.55	t = 4.55, *P* < 0.01^*a*^
Fluoroquinolone, tetracycline	2.09 ± 0.51	2.87 ± 1.70	t = 1.08, *P* = 0.32^*a*^
Glycopeptide	2.07 ± 0.35	2.87 ± 1.90	U = 0.80, *P* = 0.49^*b*^
Peptide	2.41 ± 2.38	2.46 ± 2.26	U = 0.16, *P* = 0.94^*b*^
Phenicol	2.32 ± 0.91	1.68 ± 0.84	t = 1.27, *P* = 0.23^*a*^
Macrolide, fluoroquinolone, penam	0.59 ± 0.36	3.07 ± 2.93	U = 1.44, *P* = 0.18^*b*^

a The significances were determined by Student's *t* test.

b The significances were determined by the Mann-Whitney U test.

## DISCUSSION

This study employed a metagenomics approach to investigate the effects on the fecal microbiome and ARGs among captive and free-range sika deer with the same exposure to antibiotic anthelmintics. The results revealed that (i) there were remarkable differences in the fecal microbiota composition between the captive and free-range sika deer, including taxonomic differences and overall differences based on the β-diversity; (ii) these differences in fecal microbial composition were associated with considerable shifts in microbiota function, based on KEGG, eggNOG and CAZyme annotation of the UniGenes; and (iii) although the captive and free-range sika deer had the similar antibiotic exposure, there were significant differences in AROs and antimicrobial resistance patterns.

The LEfSe, heatmap, NMDS, PCoA, and ANOSIM analyses based on the relative abundance of microbial taxa revealed noticeable differences between the two groups. The dominant microbial phyla were *Bacteroidetes* and *Firmicutes*, which is consistent with previous studies on the feces of sika deer (2, 12). However, we found that the abundance of *Bacteroidetes* was higher than the abundance of *Firmicutes*, which was opposite to the results of the above two studies. Furthermore, our results revealed that the *Firmicutes* abundance and F/B ratio were higher in the free-range deer than the captive deer, which was verified by the higher abundance of the constituent genera *Clostridium* and *Oscillibacter*, and species *Firmicutes bacterium CAG:110*. Although the members of the phyla *Bacteroidetes* and *Firmicutes* are generally dominant in the gut of humans (17) and animals (18), the proportions of these two phyla change with shifts in environmental and host factors (19). *Firmicutes* have been recognized as “obesity bacteria” because of their functions related to energy harvest and fat storage (20–22), and a higher F/B ratio has generally been associated with obesity and elevated body mass index (23, 24). The higher *Firmicutes* abundance and F/B ratio in the free-range sika deer indicated that these animals utilized their gut microbiota to derive more nutrients to support their increased activities, and the results implied better body condition. Noticeably, *Fibrobacteres* and its sole genus *Fibrobacter* both had a higher abundance in the captive deer than the free-range deer. The role of diet in modulating the animal gut microbiome is overwhelming (25), and it is generally believed that *Fibrobacter* function as a major cellulose digester to derive nutrients for herbivores (26, 27). The free-range sika deer were allowed to freely forage for vegetation in the woodland. However, the vegetation was not sufficient for the ∼100 free-range deer, which resulted in a lower fiber proportion in the diet of the free-range deer than the captive deer.

The KEGG annotation results revealed significant differences in the biological functions of the fecal microbiomes between the captive and free-range sika deer. Functions related to metabolism, environmental information, and human diseases had higher abundance in the free-range deer. This was likely because the free-range deer had more opportunities to eat more diverse types of food (especially regarding greenfeed), faced more environmental challenges, and had a greater risk of infection, which is in line with previous studies (28, 29). All the metabolism KEGG level 2 subcategories, including the metabolism of amino acids, carbohydrates, nucleotides, and xenobiotics, were more abundant in the free-range deer. This indicated the greater metabolic potential of their gut microbes to act as versatile commensals regarding the degradation of carbohydrates, amino acids, and derivatives (30). Furthermore, the remarkable shifts in the metabolic function of the fecal microbiome can be reasonably connected to the significant change in the dominant phylum *Firmicutes*.

The dominant eggNOG function was “replication, recombination, and repair”, which implied that the majority of functional activities of the gut microbiome were related to replication, growth, and fermentation (31). The high abundance of “amino acid transport and metabolism” and “nucleotide transport and metabolism” suggest that many microbes were mobilized to degrade amino acids in the feed. This indicates that the high proportion of concentrated feed in the diet of the deer. Additionally, the high abundance of “transcription” and “signal transduction mechanisms” may represent large functional potentials under environmental challenges. The CAZy database was used to annotate the carbohydrate-active enzymes, which were divided into six groups (AAs, CBMs, CEs, GHs, GTs, and PLs) encoded by the gut microbes. Among the annotated enzymes, GHs were the most common followed by GTs and CBMs. In gut microbes, GHs have crucial roles in breaking down complex carbohydrates (32) and processing various exogenous and endogenous glycoconjugates (33). The annotated enzymes CBMs are noncatalytic parts of cellulolytic enzymes and have a critical targeting function related to plant cell wall depolymerization (34, 35). The differences in GHs and CBMs between the two groups suggested that sika deer subjected to different rearing conditions utilized different gut microbes to specifically degrade diverse plant polysaccharides. All the differences in the function of the gut microbiota in sika deer between the two groups worked in concert with the differences in microbial composition, and they indicate the strong impact on the gut microbiome in captive and free-range sika deer.

Captivity and free-range are the main rearing condition for sika deer, and it is valuable to evaluate how the present practices affect environmental antibiotic resistance transmission. Unlike the composition of gut microbiota, the predominant phyla in antibiotic-resistant bacteria were *Firmicutes* and *Proteobacteria* (rather than *Firmicutes* and *Bacteroidetes*), indicating that *Proteobacteria* played a more important role in the abundance of ARGs than *Bacteroidetes* (36). We found abundant antibiotic resistances in the fecal microbiome of sika deer, and *tet*Q and *lnu*C dominated the AROs. Although the environment is important for the transmission of antibiotic resistance, these results partly confirm that antibiotic anthelmintics can induce antibiotic resistance in the gut microbiota of sika deer, which is consistent with a study on chickens (16). Although the sika deer were never fed chlortetracycline or oxytetracycline, tetracycline resistance genes were found. Due to the widespread use of tetracycline in humans and livestock, tetracycline resistance is commonly detected in feces and the environment (37, 38). According to the RDA of tetracycline resistance, *tet*Q, *tet*X, and *tet*W/N/W were related to the dominant genus *Prevotella* in the captive deer. Previous studies have reported that *tet*Q genes can be isolated from *Prevotella* strains in sick and healthy humans (36, 39). *lnu*C is a lincosamide resistance gene, which is induced by clindamycin and lincomycin (40). We found that *lnu*C in the captive group was related to the dominant genera *Paludibacter* and *Eubacterium*, which explained the higher abundance of *lnu*C AROs in the captive deer. Generally, the fecal microbiomes of the captive deer had more abundant ARGs. Because all the deer had the same exposure to antibiotic anthelmintics and basal diet and differed only in the greenfeed, breeding density, and environmental factors, we suspect that these variable factors promoted the establishment of the wide array of ARGs. Horizontal gene transfer is an important ARG transmission route, and intensive breeding accelerates the spread of ARGs and the proliferation of resistant strains (41, 42). The people, domestic animals, wildlife, plants, and environment are the basic elements in the One Health Perspective, and the spread of ARGs within and between these sectors is one of the severe threats (43). Tetracycline and lincosamide are commonly used antibiotic drugs in humans and animals, and their resistance should be considered critically.

### Conclusions.

Overall, this study provides evidence on the major effects on the gut microbiome and ARGs in captive and free-range (caused by different greenfeed diet, breeding density, and/or housing environment) sika deer. In addition, this study serves as the metagenomic sequencing study on the fecal microbiome and resistome in sika deer. The results provide essential baseline data for understanding the relationships among sika deer rearing condition, gut microbiota, and antibiotic resistance, and the conclusions can be used to evaluate the captive and free-range deer. However, the relatively small sample size and insufficient control for some potential factors (soil antibiotics, feed intake) were the main limitations of this study to draw a more robust conclusion.

## MATERIALS AND METHODS

### Animals and anthelmintic exposure.

The sampling site was a sika deer breeding center in Xiajiang county, Ji’an city, Jiangxi Province of China (27°29′N, 115°4′E). The center included a conventional captive drylot and a free-range farm with about 1 km^2^ of woodland. All sampled deer were healthy, and the information about gender, age, and weight of each animal is in Table S5. The previous antibiotic exposure was the routine albendazole-ivermectin powder treatment. For the captive group, six deer were randomly selected from the captive drylot. For the free-range group, six deer were randomly selected from the free-range farm. The captive deer were fed fresh leaves and grass during spring, summer, and autumn, and dry leaves and grass during winter. Supplementary concentrated feeds and some cooked grains were fed in all seasons. The free-range deer were fed the same concentrated feeds and cooked grains, but their greenfeed was the vegetation in the woodland. All deer were fed routine albendazole-ivermectin powder (BORY, Zhengzhou, China) in the concentrated feeds according to the manufacturer’s instructions. Both the captive and free-range deer were fed with the same water, and water was provided *ad libitum*.

### Fecal sample collection.

We collected fecal samples from deer in August 2020. The six selected captive deer were marked with ear labels so that we could distinguish individuals. These deer were separated at night, and samples from each of them were collected the next morning. Regarding the free-range deer, when we saw each of the six selected animals defecate, we collected the fresh feces immediately. The fecal samples were first preserved in a mobile refrigerator with dry ice, and then transported to the laboratory and stored at −80°C until DNA extraction.

### Extraction and assessment of fecal DNA.

The fecal samples were ground up in liquid nitrogen, and then eDNA extraction was carried out using a QIAamp DNA Stool minikit (Qiagen, Hilden, Germany) according to the manufacturer’s protocols. The DNA degradation degree and potential contamination were assessed using 1.0% agarose gel electrophoresis. Subsequently, the DNA concentration was measured using a dsDNA assay kit and a Qubit 2.0 Flurometer (Life Technologies, Carlsbad, CA, USA). Finally, DNA with an optical density (OD) value of 1.8 to 2.0 and concentration >1 μg/mL was used for library construction.

### Library construction and next-generation sequencing.

For each sample, 1 μg DNA was used as input material for the DNA sample preparation. Sequencing libraries were generated using a NEBNext Ultra™ DNA Library Prep kit for Illumina (NEB, Ipswich, MA, USA) following the manufacturer’s recommendations. Index codes were added to be able to attribute the correct sequences to the correct samples. Briefly, each DNA sample was fragmented by sonication to 350 bp. The DNA fragments were then end-polished, A-tailed, and ligated with the full-length adaptor (for Illumina sequencing) with further PCR amplification. Thereafter, the PCR products were purified, and each library’s size distribution was analyzed using an Agilent 2100 Bioanalyzer (Agilent, Santa Clara, CA, USA) and quantified using real-time PCR. Clustering of the index-coded samples was performed on a cBot Cluster Generation System (Illumina) according to the manufacturer’s instructions. Thereafter, the library preparations were sequenced on an Illumina HiSeq PE150 platform to generate paired-end (PE) reads.

### Bioinformatics and statistical analysis.

Sequencing data preprocessing was done using Readfq v8 (https://github.com/cjfields/readfq) to preprocess the raw Illumina HiSeq sequencing data. Clean data were acquired by removing low-quality reads. Given the possibility of host pollution of the samples, the clean data were subjected to a BLAST search against the host database using Bowtie v2.2.4 software (http://bowtiebio.sourceforge.net/bowtie2/index.shtml) to remove the reads of host origin (44).

Metagenome assembly was done as follows. The clean data were assembled and analyzed using SOAPdenovo v2.04 (45). The assembled Scaftigs were then interrupted from the N connection to obtain Scaftigs without N (46). All clean data were compared to each scaffold using Bowtie v2.2.4 to acquire the unused PE reads. The PE reads not used in the forward step of all samples were combined and then SOAPdenovo v2.04 was used for mixed assembly, and then break the mixed assembled scaffolds from N connection to obtain the Scaftigs. Finally, the fragments <500 bp in all Scaftigs were removed before single or mixed assembly statistical analysis.

Gene prediction and abundance analysis was performed as follows. The Scaftigs (≥500 bp) were subjected to an open reading frame (ORF) prediction using MetaGeneMark v2.10, and ORFs with <100 nt were then filtered out using default parameters (47). CD-HIT v4.5.8 was used to reduce the redundancy and obtain the unique initial gene catalog (48). The clean data of each sample was mapped to the initial gene catalog using Bowtie v2.2.4, and the number of reads mapped to each gene was calculated (49). The genes with ≤2 reads in each sample were removed to obtain the gene catalog (UniGenes) for subsequent analysis. Based on the number of mapped reads and gene length, the abundance of each gene in each sample was calculated.

Taxonomy prediction was done using DIAMOND v0.9.9 (https://github.com/bbuchfink/diamond/) to subject the UniGenes to a BLAST search against the NCBI NR database (v2018-01-02) bacterial sequences. The lowest common ancestor (LCA) algorithm in the MEGAN pipeline was used to ensure the optimum annotation of sequences (50).

Functional annotation was done using DIAMOND v0.9.9 to subject the UniGenes to BLAST searches against the KEGG (v2020-09-01), eggNOG (v5.0), and CAZy (v201901) databases. Functional hierarchy, functional annotation, and gene abundance information were then obtained.

Resistance gene annotation: Resistance Gene Identifier (RGI) software was used to align the UniGenes to the Comprehensive Antibiotic Resistance Database (CARD; https://card.mcmaster.ca/). Based on the alignment results, the relative abundance of antibiotic resistance ontology terms (AROs) was determined.

Statistical analysis and plots included linear discriminant analysis (LDA) effect size (LEfSe) using LEfSe software (default LDA score was 3) to identify differences in microbial taxa between the two groups. Heatmap construction, nonmetric multidimensional scaling (NMDS), principal-component analysis (PCA), and principal coordinate analysis (PCoA) were performed in R (http://www.r-project.org/) to visually compare the overall differences in microbial taxonomic composition, functional genes, and antibiotic resistance. Analysis of similarities (ANOSIM) and Metastats were used to acquire the *P* values of the above differences. Nonparametric permutation tests between groups were used in a redundancy analysis (RDA) to obtain a *P* value regarding the relationship between each antimicrobial resistance pattern and the main taxonomic genera. Nonparametric one-sample Kolmogorov–Smirnov tests were used to test for data normality regarding the relative abundance of taxa, functional genes, and ARGs. Student's *t* test (*t* test, for normally distributed data) and nonparametric Mann-Whitney U tests (MW test, for non-normally distributed data) were used to compare the relative abundances of taxa, functional genes, and ARGs between the two groups. The Kolmogorov-Smirnov tests, Student’s t-tests, and Mann-Whitney U tests were performed using SPSS software v24 (IBM Corporation, Armonk, NY, USA).

### Data availability.

These sequence data have been submitted to the GenBank databases with accession number PRJNA732137.
