# Supplementary material for: Differences in Fecal Microbiome and Antimicrobial Resistance between Captive and Free-Range Sika Deer under the Same Exposure of Antibiotic Anthelmintics
Source: Microbiol Spectr. 2021 Dec 1;9(3):e01918-21. doi: 10.1128/Spectrum.01918-21 (PMC8635127; doi:10.1128/Spectrum.01918-21)
Supplement: SUPPLEMENTAL FILE 1 — Supplemental material. Download SPECTRUM01918-21_Supp_1_seq9.pdf, PDF file, 0.6 MB [file spectrum01918-21_supp_1_seq9.pdf]

Table S1 The amount of raw data, clean data, and other information about the dataset obtained from 12 fecal samples of sika deer

Sample	RawData (Mbp)	CleanData (Mbp)	Clean_Q20	Clean_Q30	Clean_GC (%)	Effective (%)
C1	6,406.35	6,404.81	97.91	93.75	45.44	99.976
C2	6,299.45	6,297.90	97.75	93.49	45.91	99.975
C3	6,231.93	6,231.25	97.61	93.12	45.52	99.989
C4	6,005.46	6,003.94	97.67	93.29	45.24	99.975
C5	6,016.91	6,016.23	97.50	92.95	45.59	99.989
C6	6,492.01	6,489.84	97.41	92.84	46.09	99.967
F1	6,842.55	6,839.32	97.84	93.66	44.54	99.953
F2	6,421.30	6,420.32	97.16	92.23	45.30	99.985
F3	6,726.79	6,724.52	97.67	93.28	45.17	99.966
F4	6,024.75	6,023.39	97.55	93.06	44.99	99.977
F5	6,527.61	6,524.20	97.68	93.30	44.95	99.948
F6	6,378.66	6,372.23	97.85	93.73	47.33	99.899

Table S2 The length (bp) and number of scaftigs obtained from 12 fecal samples of sika deer

Sample	Total length	Scaftigs Number	Average length	N50 length	N90 length	Max length
C1	140,373,821	125,135	1,121.78	1,167	566	182,068
C2	145,095,631	114,762	1,264.32	1,410	577	248,282
C3	154,706,817	122,988	1,257.90	1,421	577	449,244
C4	173,830,585	128,203	1,355.90	1,614	588	397,036
C5	145,842,452	115,224	1,265.73	1,451	579	406,375
C6	120,240,736	102,699	1,170.81	1,288	572	166,066
F1	174,690,084	141,424	1,235.22	1,373	579	196,138
F2	150,948,233	117,981	1,279.43	1,513	587	135,317
F3	118,846,448	98,224	1,209.95	1,320	570	270,899
F4	153,503,757	127,193	1,206.86	1,334	578	186,600
F5	171,279,432	134,847	1,270.18	1,433	581	205,052
F6	155,879,344	127,625	1,221.39	1,351	583	318,988

Table S3 The number, length, and other information about predicted Open Reading Frames (ORFs) obtained in 12 fecal samples of sika deer

Samples	ORFs number	[1]integrity: none (percentage %)	[2]integrity: end (percentage %)	[3]integrity: start (percentage %)	[4]integrity: all (percentage %)	Total length (Mbp)	Average length (bp)	GC percent (%)
C1	211,919	30,266(14.28%)	50,146(23.66%)	67,741(31.97%)	63,766(30.09%)	126.07	594.91	46.74
C2	208,911	26,910(12.88%)	46,959(22.48%)	62,593(29.96%)	72,449(34.68%)	129.95	622.01	46.03
C3	221,067	29,968(13.56%)	49,805(22.53%)	66,334(30.01%)	74,960(33.91%)	139.89	632.80	46.10
C4	240,880	30,460(12.65%)	52,783(21.91%)	69,464(28.84%)	88,173(36.60%)	156.59	650.09	45.62
C5	207,620	27,783(13.38%)	46,911(22.59%)	62,290(30.00%)	70,636(34.02%)	131.92	635.38	46.23
C6	176,478	23,558(13.35%)	41,423(23.47%)	56,041(31.76%)	55,456(31.42%)	107.37	608.40	45.60
F1	254,186	31,351(12.33%)	58,370(22.96%)	79,767(31.38%)	84,698(33.32%)	156.12	614.18	45.80
F2	216,106	25,324(11.72%)	49,834(23.06%)	67,203(31.1%)	73,745(34.12%)	135.14	625.35	45.63
F3	176,611	22,842(12.93%)	39,953(22.62%)	53,548(30.32%)	60,268(34.12%)	106.34	602.11	45.06
F4	224,708	28,311(12.6%)	52,648(23.43%)	71,109(31.65%)	72,640(32.33%)	137.16	610.38	46.10
F5	249,597	28,962(11.6%)	56,462(22.62%)	76,869(30.8%)	87,304(34.98%)	152.42	610.66	45.82
F6	229,110	27,454(11.98%)	52,406(22.87%)	72,410(31.6%)	76,840(33.54%)	139.64	609.49	48.07

Table S4

The within and between sampling group Bray–Curtis similarity. Free, free-range sika deer; Captive, captive sika deer.

Within or between group	Bray–Curtis similarity (Mean ± SD)	Significance
Free	0.89 ± 0.04	
Captive	0.90 ± 0.04	F=1.76, P=0.18
Free-Captive	0.88 ± 0.02	

Table S5 The gender, age, and weight of sampled sika deer. C1-C6, captive sika deer; F1-F6, free-range sika deer.

Individuals	Gender	Age (years)	Weight (kg)
C1	Male	6	80.20
C2	Male	6	80.50
C3	Male	6	79.60
C4	Male	6	81.30
C5	Male	6	81.50
C6	Male	6	82.10
F1	Male	6	81.40
F2	Male	6	80.20
F3	Male	6	79.90
F4	Male	6	81.30
F5	Male	6	81.60
F6	Male	6	78.90

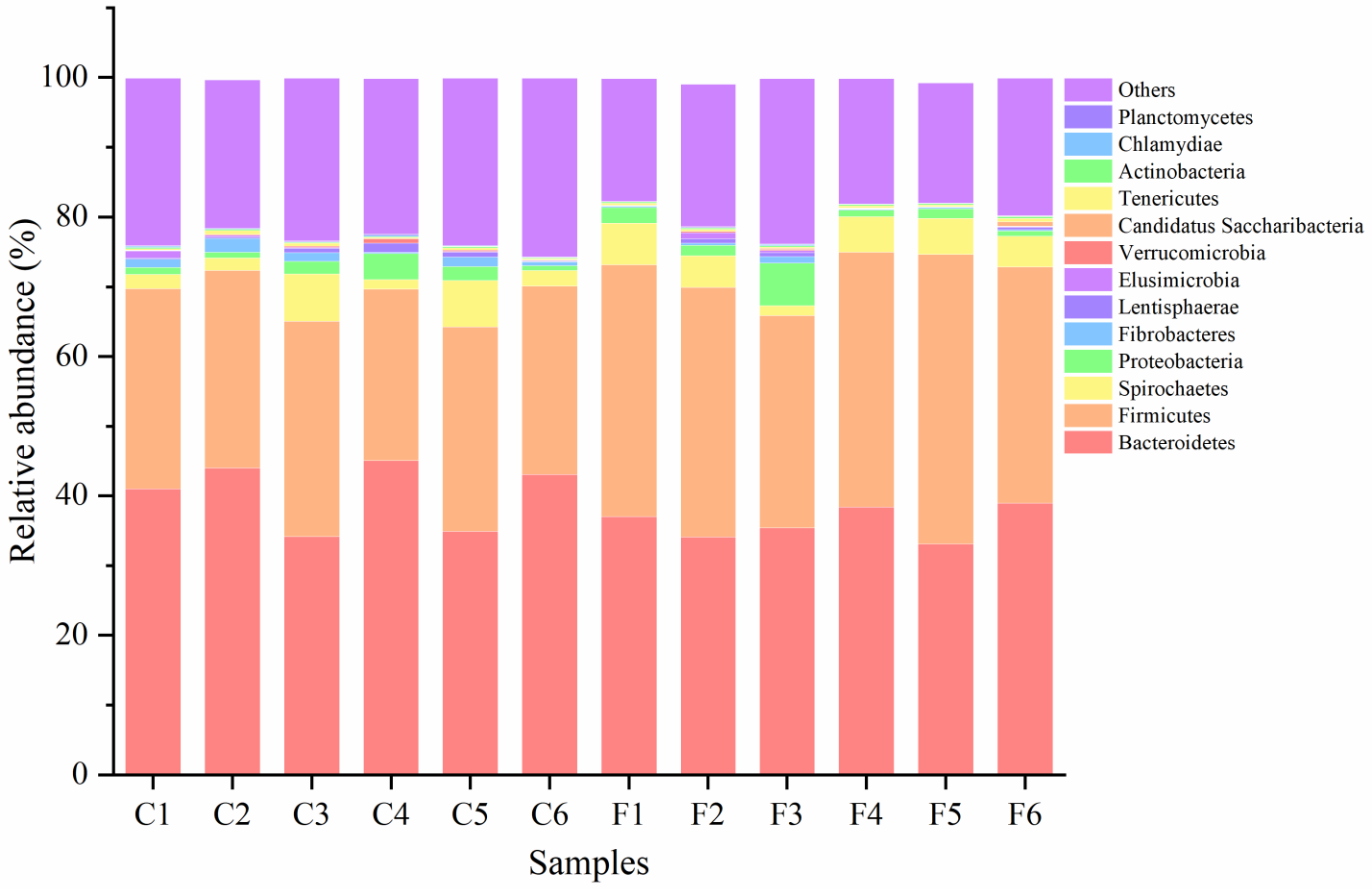

Figure S1 The relative abundance of microbial phyla in fecal microbiome of captive (Captive, C1-C6) and free-range (Free, F1-F6) sika deer. The sequences with low mean relative abundance (<0.1%) were assigned as "Others".

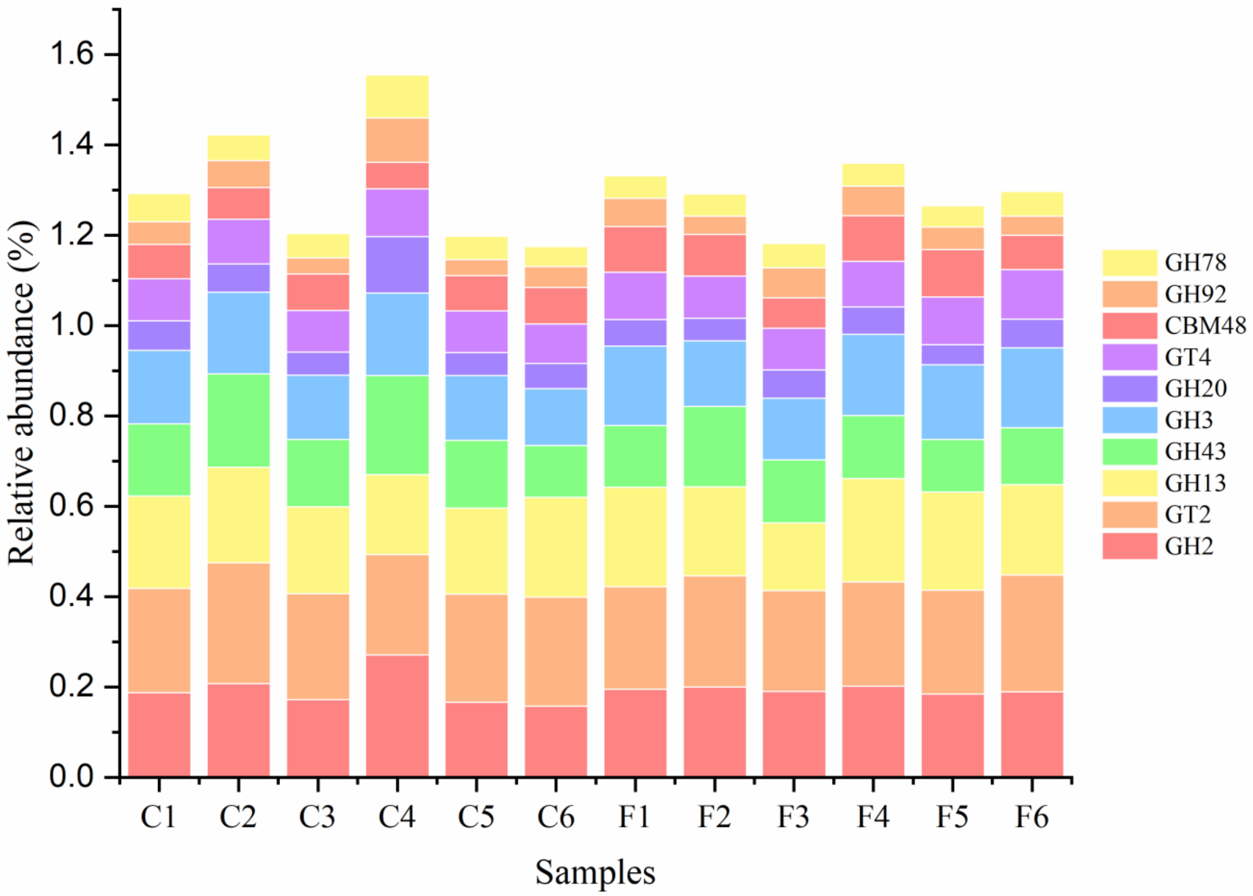

Figure S2 The relative abundance of top 10 CAZymes in microbial function of captive (Captive, C1-C6) and free-range (Free, F1-F6) sika deer. GH represents glycoside hydrolase, CBM represents carbohydrate-binding module, GT represents glycosyl transferase.

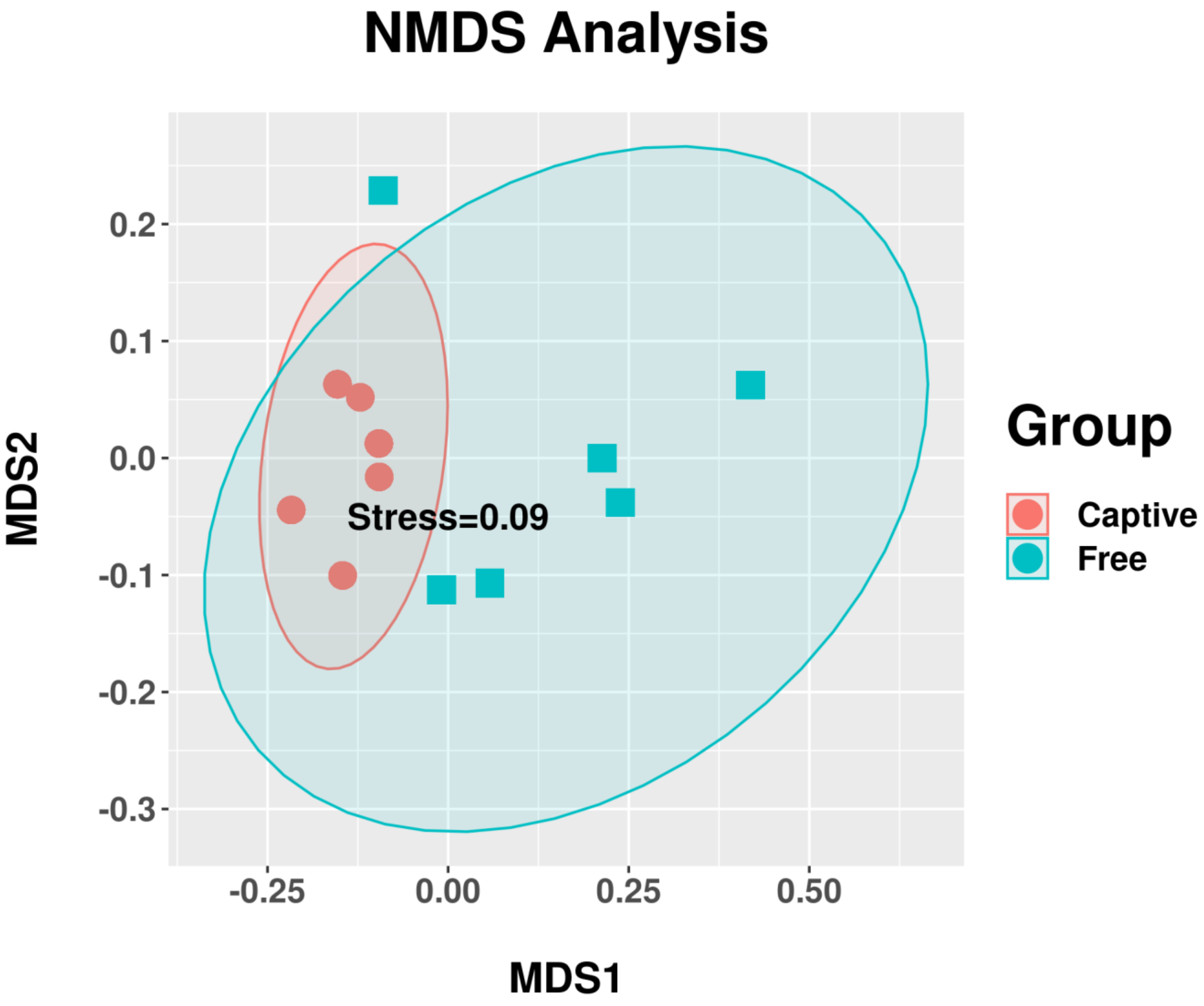

Figure S3 Non-metric Multidimensional Scaling plots (B, NMDS, based on Bray-Curtis dissimilarities of relative abundance of antibiotic resistance ontologies) shows the differences in antibiotic resistance of fecal microbiome in captive (Captive) and free-range (Free) sika deer.

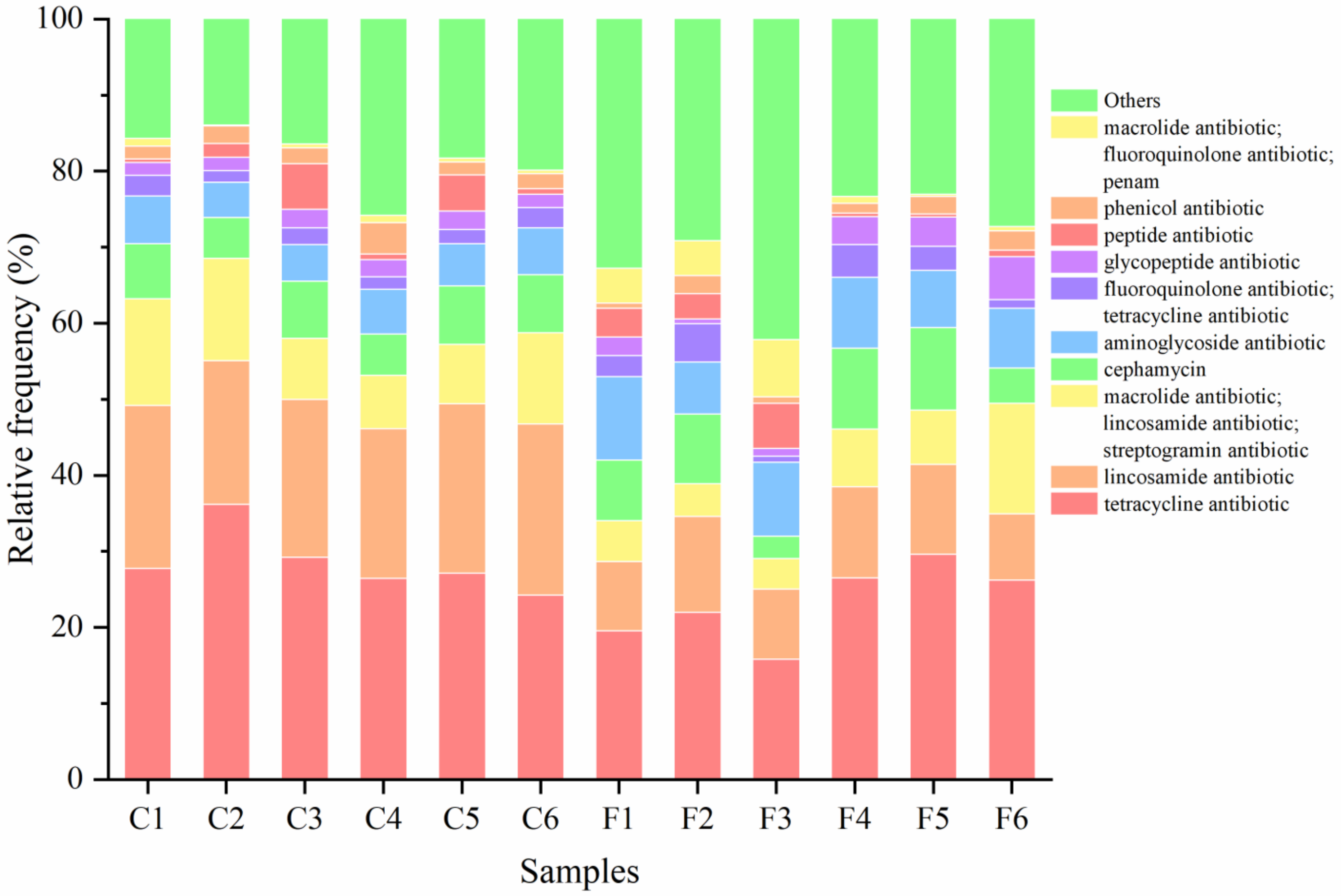

Figure S4 The relative abundance of antimicrobial resistance patterns in fecal microbiome of captive (Captive, C1-C6) and free-range (Free, F1-F6) sika deer.
